# Comparison of 4D flow and 2D PC MRI blood flow quantification in children and young adults with congenital heart disease

**DOI:** 10.1186/1532-429X-15-S1-E90

**Published:** 2013-01-30

**Authors:** Maya Gabbour, Cynthia Rigsby, Michael Markl, Susanne Schnell, Kelly B Jarvis, Roger A de Freitas, Andrada R Popescu, Joshua D Robinson

**Affiliations:** 1 Department of Medical Imaging, Ann & Robert H Lurie Children's Hospital of Chicago, Chicago, IL, USA; 2 Department of Radiology, Northwestern University Feinberg School of Medicine, Chicago, IL, USA; 3 Department of Biomedical Engineering, Northwestern University, Chicago, IL, USA; 4 Division of Pediatric Cardiology, Ann & Robert H Lurie Children's Hospital of Chicago, Chicago, IL, USA; 5 Department of Pediatrics, Northwestern University Feinberg School of Medicine, Chicago, IL, USA

## Background

Echocardiography (echo) is the primary imaging modality for assessment of aortic and pulmonary blood flow velocities. 2D phase contrast (PC) MRI provides better access to all segments of the aortic and pulmonary system and is considered the standard for evaluating blood flow. Both techniques are limited by velocity analysis in 2D planes and by single-direction velocity measurement which may be inadequate to characterize the complex 3D hemodynamics in congenital heart disease (CHD). 4D flow MRI provides simultaneous assessment of 3D blood flow characteristics of all vessels within a 3D volume and offers the ability to retrospectively quantify blood flow parameters at selectable regions of interest. The aim of this study is to test the potential of 4D flow for accuracy of quantification of aortic and pulmonary flow parameters compared to the reference standards echo and 2D PC MRI in children and young adults with CHD.

## Methods

32 patients with CHD who underwent simultaneous 4D flow and 2D PC MRI and echo within 9 months of MRI were retrospectively included. 2D PC MRI flow quantification in the aortic root (Ao), pulmonary trunk (PT), and right and left pulmonary arteries (RPA, LPA) was analyzed using Medis (Medis, Leiden, The Netherlands). 4D flow data analysis included calculation of a 3D-PC-angiogram which was used to position analysis planes in the Ao, PT, LPA and RPA (EnSight, CEI, Apex, NC) for quantification of net flow, regurgitant fraction, Qp:Qs, and peak velocities. Ao peak velocities were assessed by echo. Linear regression analysis was performed. Pearson's correlation coefficient (r) was calculated. A correlation with p<0.05 was considered significant.

## Results

Patient characteristics are listed in Table 1. Mean time between MRI and echo was 2.7 months. Excellent agreement was found between 4D flow and 2D PC MRI for quantification of net flow (r=0.95, p<0.001) and regurgitant fraction (r=0.91, p<0.001) in the Ao, PT, RPA and LPA (Figure 1). For peak velocities, a significant but more moderate relationship (r=0.46, p<0.001, Figure 1) between 4D flow and 2D PC MRI was found. After excluding patients with shunts (n=2), Fontan circulation (n=3), and with incomplete 2D PC MRI data (n=6), Qp:Qs showed good agreement between 4D flow and 2D PC MRI (r=0.63, p=0.001). Noticeably, Qp:Qs based on 4D flow MRI showed a better approximation of the expected ratio of 1 (0.98 for 4D flow vs. 0.93 for 2D PC MRI). For aortic peak velocities, both 2D PC and 4D flow MRI demonstrated good and similar agreement with echo (r=0.58, p<0.003 and r=0.55, p=0.005, respectively)

**Table 1:** Patient population characteristics

PT#	Age (yrs)	Diagnosis
1	14	TOF, post repair
2	6	TOF, post repair
3	20	TOF, post repair
4	10	TOF, post repair
5	7	TOF, post repair
6	6	TOF, post repair
7	11	TOF/absent pulmonary valve, post repair
8	14	TOF, AVSD, post repair with RV to PA conduit, RPA stent
9	10	TOF/pulmonary atresia, post Rastelli and right unifocalization
10	11	TOF/pulmonary artresia, post repair with RV to PA conduit
11	5	TOF/pulmonary atresia, post bilateral unifocalization and RV to PA conduit
12	5	d-TGA post arterial switch
13	16	d-TGA post arterial switch
14	6	d-TGA post arterial switch
15	7	d-TGA post arterial switch
16	29	cc-TGA
17	28	BAV, post Ross
18	10	BAV, post Ross
19	16	Aortic coarctation, post subclavian flap repair
20	7	Severe unrepaired aortic coarctation
21	11	Ventricular ectopy
22	14	ASD, VSD post repair with pulmonary valve stenosis
23	12	VSD and pulmonary stenosis post repair, vascular ring
24	5	LV non-compaction
25	10	Truncus ateriosus, post repair with RV-PA conduit
26	20	d-TGA, tricuspid atresia, pulmonary stenosis, s/p lateral tunnel Fontan
27	21	DORV, mitral atresia, post fenestrated lateral Fontan
28	9	Pulmonary atresia, post Fantan
29	19	Williams syndrome and subaortic stenosis, post resection of subaortic fibrous ring
30	26	Castleman's syndrome and subaortic stenosis, post resection of subaortic fibrous ring
31	12	Mild hyoplasia of the distal transverse aortic arch
32	16	Heart murmur

Abbreviation key: TOF, tetralogy of Fallot; AVSC, atrioventrincular septal defect; RV, right ventricle; PA, pulmonary artery; RPA, right pulmonary artery; d-TGA, d-transposition of the great arteries; cc-TGA, congenitally corrected transposition of the great arteries; BAV, bicommisural aortic valve; ASD, atrial septal defect; VSD, ventricular septal defect; LV, left ventricle; DORV, double outlet right ventricle.

## Conclusions

Flow quantification based on 4D flow MRI showed good-excellent correlation for clinically relevant flow parameters for the characterization of CHD such as pulmonary and aortic peak velocities, net flow, regurgitant fraction and Qp:Qs compared to the references standards 2D PC MRI and echo.

## Funding

Grant support NIH R01HL115828 and NUCATS Dixon Award

